# Toward precision host-directed immunomodulation in community-acquired pneumonia: a proposed endotype–target–natural-product framework and a roadmap for validation in human lung organoids

**DOI:** 10.3389/fmed.2026.1941520

**Published:** 2026-09-28

**Authors:** Qing Shen, Fengjun Zhang, Fan Wu, Yilin Wang, Zijian Su, Guihua Xu, Xing Zhang, Wei Zhang

**Affiliations:** 1 Department of Pulmonary Diseases, Shuguang Hospital Affiliated to Shanghai University of Traditional Chinese Medicine, Shanghai, China; 2 Department of Pulmonary Diseases, Shuguang Hospital, Shanghai Institute of Infectious Diseases and Biosecurity, Shanghai University of Traditional Chinese Medicine, Shanghai, China; 3 Zhang Wei Baoshan Famous Traditional Chinese Medicine Inheritance Studio, Shanghai Baoshan Hospital of Integrated Chinese and Western Medicine, Shanghai, China; 4 Institute of Infectious Diseases, Shanghai Institute of Traditional Chinese Medicine, Shanghai, China

**Keywords:** community-acquired pneumonia, host-directed therapy, inflammatory endotype, lung organoids, natural products, neutrophil extracellular traps

## Abstract

Community-acquired pneumonia (CAP) still causes high mortality despite effective antibiotics. Much of this death is driven by a dysregulated host immune response rather than by the pathogen itself, and antimicrobial resistance deepens the problem. Host-directed therapy has accordingly shifted toward precision immunomodulation, yet its benefit appears conditional on the inflammatory state. Stratification by a single inflammator*y* axis such as C-reactive protein (CRP) separates broad prognostic subgroups, but it does not indicate which effector pathway to target or which agent to use; we designate this limitation, as a conceptual shorthand rather than a validated threshold, the “CRP ceiling.” Existing reviews treat natural-product host-directed therapy, CAP inflammatory endotyping, and human lung organoid modeling separately; the gap we address is the absence of a framework that connects them. Integrating these three fields, we propose, as a hypothesis-generating rather than a validated scheme, an endotype–target–natural-product framework for bacterial CAP. We first outline the interventable core of CAP immunopathology across the inflammasome, neutrophil extracellular trap (NET), and resolution axes, together with the pathogen-specific virulence that biases them; we then propose that the multi-target pharmacology of natural products may suit candidate endotypes driven by several co-activated nodes, where single-target agents have often failed, while noting that their comparatively favorable toxicity is itself a hypothesis rather than an established class property; and we position human lung organoids as a promising human-relevant platform, alongside complementary systems, that could test endotype-, compound-, and pathogen-specific hypotheses and provide a roadmap for validation before clinical translation. The framework is intended to complement, not replace, pathogen-directed treatment, and to reframe host-directed therapy from uniform anti-inflammation toward modulation matched to mechanism, pathogen, and patient. The principal challenges concern immune-competent organoids, validated pathway-level endotype biomarkers, and standardized characterization of multi-component natural products.

## 1 Introduction

Community-acquired pneumonia (CAP) remains the most persistent infectious cause of death worldwide, and its burden is now compounded by antimicrobial resistance (AMR). The 2024 Global Burden of Disease analysis identified lower respiratory infection as the single largest contributor to AMR-associated mortality, accounting for roughly 1.5 million of the 4.7 million deaths associated with bacterial resistance in 2021 (1). Non-COVID-19 lower respiratory infections caused an estimated 2.2 million deaths in the same year, falling disproportionately on older adults and young children (2). Against this backdrop, the pathogens that dominate bacterial CAP and its viral-bacterial complications, including *Streptococcus pneumoniae*, *Staphylococcus aureus*, *Pseudomonas aeruginosa*, and influenza-associated secondary infection, continue to drive severe disease even where potent antibiotics are available (3, 4). The viral and bacterial elements of this spectrum are also intertwined: antecedent influenza predisposes to secondary pneumococcal and staphylococcal pneumonia through lasting effects on alveolar immune cells (5). This paradox, in which effective bactericidal therapy fails to prevent death, points to a determinant of outcome that lies in the host rather than the microbe.

Much of the mortality in severe CAP is attributable to a dysregulated host response, in which pathogen recognition triggers NF-*κ*B activation, NLRP3 inflammasome assembly, neutrophil extracellular trap (NET) release, and a failure of inflammation resolution, together disrupting the alveolar-capillary barrier (6, 7). This understanding has motivated host-directed therapy (HDT), a strategy that modulates the immune response to limit tissue injury or improve pathogen clearance rather than acting on the pathogen itself (8). HDT is especially attractive in the MDR era, because it imposes no direct selective pressure on bacteria and may remain effective as antibiotic options narrow (9, 10). It is, however, an adjunct: bacterial control and source control remain essential, and HDT is intended to complement rather than replace pathogen-directed treatment (8). Host-directed therapy is not a single tactic but a spectrum, ranging from dampening a specific injurious pathway to boosting a defective effector function, and its safe use depends on knowing which of these a given patient needs (8). This dependence on host state, rather than on the drug alone, makes precise patient selection the central problem for the field.

Progress in HDT has converged on a precision approach built around inflammatory endotypes. The CAPE COD trial showed that adjunctive hydrocortisone reduces 28-day mortality in patients admitted to intensive care with severe CAP (11), and secondary analyses suggest that this benefit concentrated in patients with pronounced systemic inflammation (12, 13). This pattern is most cautiously read as heterogeneity of treatment effect (evidence that inflammatory context modifies the response, and a rationale for predictive enrichment) rather than as proof that a validated endotype-to-drug rule already exists (12). Latent class analyses have repeatedly resolved CAP into a hyperinflammatory and a hypoinflammatory subgroup with distinct outcomes (13, 14). The granularity at which stratification stays useful is nonetheless uncertain: within an already hyperinflammatory population, data-driven sub-phenotyping did not modify the response to corticosteroids (15). We use the term “CRP ceiling” throughout as a conceptual shorthand for this limit: the point at which a single summary inflammator*y* axis such as CRP separates broad subgroups but can no longer guide finer therapeutic choices. It denotes a practical boundary of current single-axis stratification, not a demonstrated biological threshold, and CRP-based enrichment should be distinguished from the broader latent-class, transcriptomic, or multi-omic phenotyping that may eventually refine it. This limit defines the central problem this review addresses.

The difficulty is not a shortage of candidate interventions but a shortage of criteria for deploying them. Corticosteroids, colony-stimulating factors, statins, inflammasome inhibitors, and natural products have each shown host-protective activity in some settings and none in others, a pattern that reflects unselected use more than intrinsic failure (8, 12). A route through this problem needs two things the field has pursued separately: a way to define which host process is driving injury in a given patient, and agents whose breadth of action fits that process.

Natural products offer a mechanistically distinct way through this deadlock. Compounds from the flavonoid, polysaccharide, terpenoid, and polyphenol classes act on several nodes of the injurious host response at once, including NLRP3, TLR4/NF-*κ*B, NET formation, and macrophage polarization, and, in some settings, do so with a lower toxicity burden than single-target synthetic inhibitors; the hepatotoxicity that halted clinical development of the NLRP3 inhibitor MCC950 illustrates the contrast (16, 17).

This comparison is a hypothesis to be tested, not an established class property, because natural-product safety is compound- and formulation-specific and several agents carry their own dose-dependent liabilities (18). Traditional formulas and their constituents have accumulated preclinical and clinical evidence in pneumonia and acute lung injury, including a randomized trial of XueBiJing in severe CAP (19) and mechanistic studies of andrographolide, astragaloside, and baicalin (20–22). Two limitations persist. Most evidence derives from viral pneumonia or endotoxin-driven injury models rather than bacterial CAP endpoints (23), and no natural product has yet been matched to a defined inflammatory endotype or validated in a human-relevant platform. Human lung organoids, which reconstruct airway and alveolar epithelium and have been used to screen antimicrobial and natural-product candidates, are one of several human-relevant systems that could supply supply the missing experimental bridge (24, 25).

This review integrates three fields that have so far advanced separately, namely natural-product HDT, CAP inflammatory endotyping, and human lung organoid modeling, into a single framework for bacterial CAP. Existing reviews treat these strands individually: traditional medicine for viral pneumonia (26), corticosteroid stratification by phenotype (12), or organoid infection models for tuberculosis and SARS-CoV-2 (27); none connects a mechanism-based endotype to a mechanistically matched natural product and to a human platform in which that match could be tested. We instead propose an “endotype-target-natural product” matching scheme, ask where the multi-target pharmacology of natural products might extend stratification beyond CRP ceiling, and outline how organoid platforms and complementary human systems can test these hypotheses before clinical translation. Throughout, the framework is advanced as hypothesis-generating: it organizes existing evidence into testable predictions rather than asserting a validated clinical paradigm. We first dissect the immunopathology of CAP, then examine inflammatory endotypes, natural-product immunomodulators, their endotype matching, and the organoid platforms that can validate them.

### 1.1 Literature search and evidence selection

This is a narrative, integrative review rather than a systematic review, and its conclusions should be read accordingly. We searched PubMed and Web of Science, together with preprint servers, for English-language articles published up to 30 June 2026, combining the concepts community-acquired pneumonia, acute lung injury, host-directed therapy, inflammatory endotype/subphenotype, inflammasome, neutrophil extracellular traps, inflammation resolution, natural products/traditional Chinese medicine, and lung organoid/organ-on-chip. Reference lists of key articles were screened manually to capture landmark and recent work; other databases (e.g., Cochrane, CNKI, Embase) were not systematically searched, consistent with this review's narrative rather than systematic scope. Because the field spans human trials, animal and *in vitro* mechanistic studies, and emerging model systems, we did not restrict inclusion to a single study design; instead we prioritized (i) randomized and controlled human CAP evidence, followed by (ii) mechanistic studies in bacterial infection models, and (iii) supporting evidence from viral, endotoxin, or other acute-lung-injury models, and we weighted conclusions toward the most direct evidence available. Preprints are included where they fill a specific gap and are labeled as such in the text and figures. This selection is illustrative of the arguments developed here rather than exhaustive, and selective citation is an acknowledged limitation of the narrative format.

## 2 The immunopathology of CAP: from pathogen killing to host injury

The tissue damage that determines outcome in severe CAP is generated largely by the host's own innate immune response, whose effector programs protect the airspace when proportionate and destroy it when excessive. Pattern recognition of invading pathogens sets in motion inflammasome activation, neutrophil recruitment with extracellular trap release, and a macrophage-directed resolution program; the balance among these processes, rather than the presence of the organism alone, separates self-limited pneumonia from acute lung injury. Each effector arm is double-edged, and each is tuned by pathogen-specific virulence. A single anti-inflammatory manipulation can therefore help or harm. The outcome depends on timing, magnitude, and the causative organism. Mapping where this response becomes maladaptive defines the interventable substrate for host-directed therapy (Figure 1).

**Figure 1:**
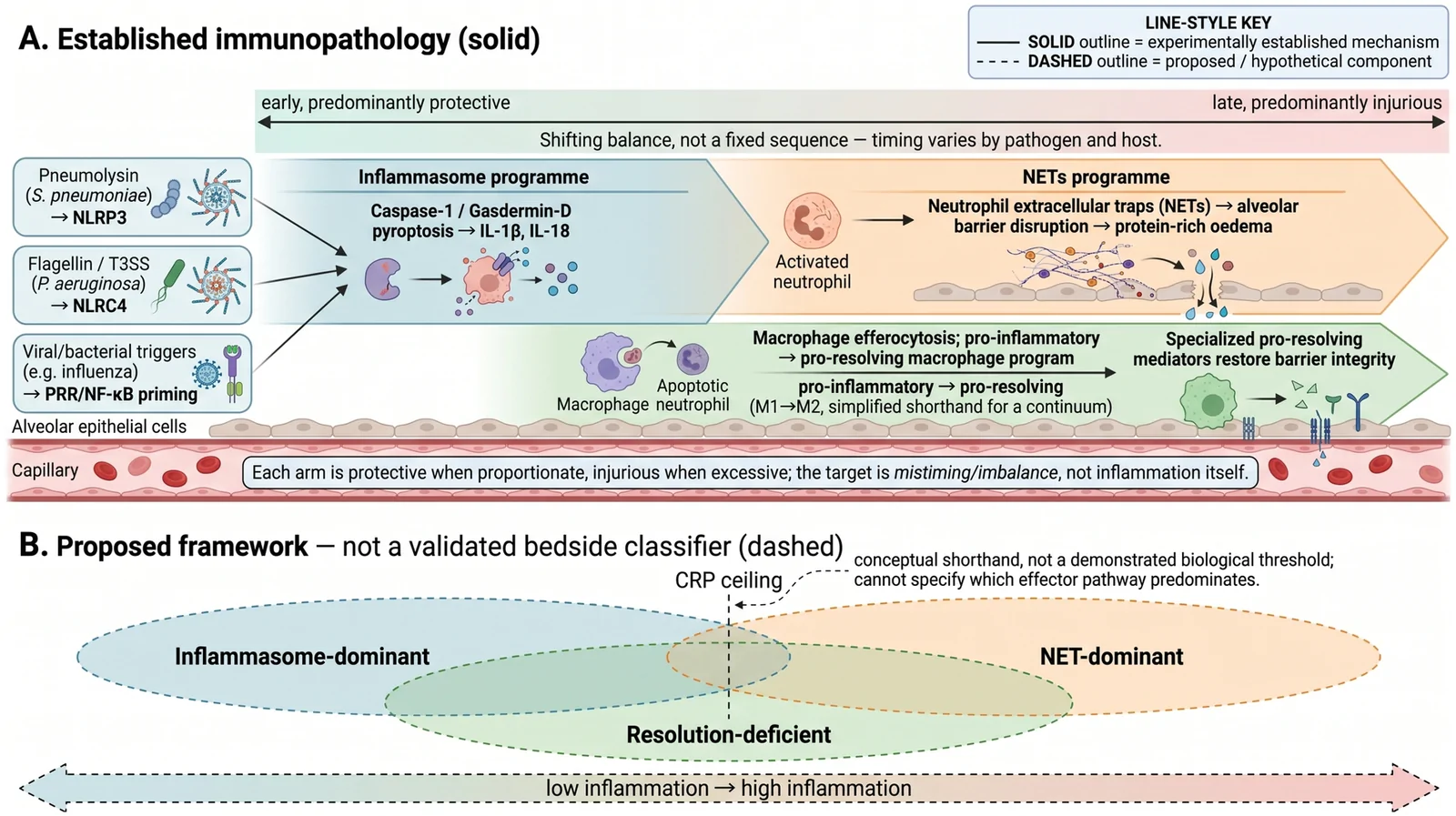
From pathogen recognition to host injury: the effector axes of CAP immunopathology and their partition into proposed inflammatory endotypes. **(A)** Along a temporal dimension from early, predominantly protective to late, predominantly injurious responses, three innate-immune effector programmes are shown across the alveolar epithelial and alveolar-capillary barrier. The programmes overlap rather than follow discrete phases: resolution can begin early, and protective and injurious functions coexist, so the left-to-right arrangement denotes a shifting balance rather than a fixed biological sequence, and its timing varies by pathogen and host. Pathogen recognition is pathogen-biased: pneumolysin from *Streptococcus pneumoniae* activates NLRP3, flagellin and the type III secretion system of *Pseudomonas aeruginosa* engage NLRC4, and viral or bacterial triggers (e.g., influenza) drive PRR/NF-*κ*B priming (6, 28, 32). Effector injury proceeds through caspase-1- and gasdermin-D-dependent pyroptosis with release of IL-1β and IL-18, and through neutrophil extracellular traps (NETs) that disrupt the alveolar barrier and produce protein-rich oedema (6, 29, 37). Resolution is mediated by macrophage efferocytosis, a shift from a pro-inflammatory to a pro-resolving macrophage program (labeled M1-to-M2 as a simplified experimental shorthand for a continuum of states), and specialized pro-resolving mediators that restore barrier integrity (44, 46). Each arm is protective when proportionate and injurious when excessive; the interventable substrate is therefore the mistiming and imbalance of these programmes rather than inflammation itself. **(B)** The three mechanistic nodes map onto three proposed inflammatory endotypes (inflammasome-dominant, NET-dominant, and resolution-deficient) overlaid on the clinical high- vs. low-inflammation gradient. These endotypes are candidate, potentially overlapping states rather than validated discrete categories. The C-reactive protein (CRP) axis separates broad inflammatory phenotypes but reaches a practical limit (the “CRP ceiling”, a conceptual shorthand rather than a demonstrated biological threshold) that cannot specify which effector pathway predominates (13). The upper mechanism tier represents established immunopathology; the lower endotype tier is a proposed framework, not a validated bedside classifier. Created in BioRender. Zhang, F. (2026) https://BioRender.com/2irpwkg

Inflammasome signaling occupies the first decisive node between defense and damage. Recognition of bacterial products primes NF-*κ*B-dependent transcription of pro-IL-1β and pro-IL-18, after which sensors such as NLRP3 and NLRC4 assemble to activate caspase-1, mature these cytokines, and trigger gasdermin D-dependent pyroptosis (6). In pneumococcal infection the pore-forming toxin pneumolysin is a principal NLRP3 activator, and controlled inflammasome activity supports early bacterial containment and alveolar-barrier maintenance, a protective function that can operate independently of IL-1β through increased epithelial adherence (28, 29). The same pathway becomes injurious when activation is disproportionate: excessive pyroptosis releases alarmins that amplify neutrophilic inflammation, and inflammasome tone shifts with host state, as the decline of NLRP3 activity in the aged lung raises susceptibility to secondary pneumococcal disease (30, 31). For Gram-negative CAP, flagellin and the type III secretion apparatus of *P. aeruginosa* engage NLRC4 to drive caspase-1-dependent IL-1β release and pyroptosis in both macrophages and neutrophils (32, 33). This response clears organisms, yet it can precipitate destructive inflammation and, in some settings, impair macrophage phagocytosis (34).

The downstream consequences of this signaling determine barrier integrity. Mature IL-1β and IL-18 amplify neutrophil recruitment and endothelial activation, while gasdermin D pores execute pyroptosis and release alarmins that draw in further immune cells (6). When the pore-forming programme runs unchecked, the alveolar epithelium loses the adherence that NLRP3 otherwise supports, and protein-rich fluid floods the airspace (29). The executioner step of gasdermin-mediated lysis therefore sits at the junction between controlled cytokine maturation and destructive membrane rupture, and interventions that tune rather than abolish this axis are more promising than complete blockade (35).

Neutrophil extracellular traps extend this defense-damage duality into the airspace. NETs immobilize and kill bacteria, and their deficient formation or premature degradation predisposes to invasive infection (36). Abundant or poorly cleared NETs, by contrast, correlate with alveolar barrier disruption: in murine MRSA and *P. aeruginosa* pneumonia, NET burden tracks with lung permeability, and limiting NET formation through PAD4 inhibition or dismantling existing traps with DNase reduces injury without abolishing bacterial control (7, 37). Pathogen-specific pathways shape this outcome, with CLEC5A driving *P. aeruginosa*-induced NET formation and lung injury, and *S. aureus* deploying nucleases and surface factors to evade trap killing (38, 39). The therapeutic logic of NET modulation has reached the clinic, where nebulized dornase alfa lowered inflammation and shortened recovery in severe viral pneumonia, evidence that extracellular chromatin is an actionable target across hyperinflammatory pneumonias (40). This evidence is drawn from viral (COVID-19) rather than bacterial CAP, and whether NET dismantling preserves bacterial clearance in pyogenic pneumonia remains the open question that any NET-directed strategy must answer (7, 40). Beyond direct cytotoxicity, the chromatin scaffold of NETs promotes local coagulation and microvascular obstruction, which couples inflammation to thrombosis in the injured lung (7).

Whether inflammation resolves depends on macrophage functional state and on the mode of neutrophil death. Alveolar macrophages clear apoptotic neutrophils and debris through efferocytosis, and impaired efferocytosis sustains inflammation and injury (41, 42). The death program of the neutrophil governs this step: gasdermin-E-driven pyroptosis provokes inflammation, whereas skewing neutrophils toward apoptosis augments macrophage efferocytosis and alleviates experimental lung injury (35). Shifting macrophages from a pro-inflammatory toward a pro-resolving, efferocytic program accelerates clearance, an axis that specialized pro-resolving mediators exploit; aspirin-triggered resolvin D1 and related lipoxins improve bacterial clearance by promoting a pro-resolving macrophage phenotype and phagocytosis rather than by direct antibacterial action, and blockade of their shared receptor worsens pneumococcal lung injury (43–45). Resolution is therefore an active, druggable program whose failure, not merely the intensity of the initial insult, perpetuates damage (46).

The pathogens that dominate CAP each bias this host response through distinct virulence strategies. Pneumolysin contributes to lung injury and epithelial apoptosis while also suppressing the initial macrophage cytokine response through SOCS1 to evade early immunity, so that its net inflammatory effect is context-dependent (47, 48). This duality means that an anti-inflammasome strategy must be timed to the phase in which pneumolysin drives damage, not the phase in which residual inflammasome activity still aids containment (47). *P. aeruginosa* tunes neutrophil pyroptosis through the exotoxins ExoU and ExoS, and it blocks non-canonical inflammasome signaling to establish persistence (49). *Klebsiella pneumoniae*, increasingly hypervirulent and carbapenem-resistant, relies on capsule-based evasion, and clearance of clinical strains depends on neutrophils and CCR2⁺ monocytes coordinated by IL-17 (50, 51). *Staphylococcus aureus* injures the airway through pore-forming and secreted-protease strategies while remaining dependent on neutrophil-mediated clearance, so that its subversion of neutrophil killing both delays bacterial control and prolongs damaging inflammation (52, 53). Antecedent influenza compounds these mechanisms by impairing alveolar macrophage function and predisposing to secondary bacterial pneumonia, linking the viral and bacterial elements of the CAP pathogen spectrum (5). These organism-specific patterns are biases that shift the probability of a given effector state, not deterministic rules that assign one pathogen to one endotype; host age, comorbidity, inoculum, co-infection, and prior antibiotics all modify the response. These organism-specific patterns help explain why uniform immunomodulation yields inconsistent results, and they argue for interventions matched to the dominant injury pathway as measured in the host.

Viewed across these effector arms, the interventable core of CAP immunopathology is not inflammation itself but its mistiming and disproportion: early inflammasome and NET activity that secures pathogen control gives way to a later, resolution-deficient phase that drives barrier failure. These phases overlap rather than follow a clean sequence; resolution programs can begin early, and protective and injurious functions coexist. The temporal picture is therefore one of shifting balance rather than discrete stages (41, 46). The principal knowledge gaps concern this temporal dimension and its pathogen dependence, namely when suppression becomes safe, which effector to target for a given organism, and how to preserve clearance while limiting damage. Meeting these needs calls for interventions that act on several nodes with tunable timing rather than on a single mediator, and for experimental systems that resolve human, pathogen-specific responses. Both requirements frame the endotype-based stratification and the multi-target natural-product strategies examined in the sections that follow.

## 3 Inflammatory endotypes and the case for precision immunomodulation

The clinical failure of uniform anti-inflammatory treatment in CAP reflects a biological reality: patients differ in the character of their host response, and not simply in its severity, and the benefit of immunomodulation is conditional on that response. Recognizing this heterogeneity has reframed adjunctive therapy as a problem of stratification rather than of average effect, and has produced reproducible inflammatory subgroups with divergent outcomes and treatment responses. The unresolved question is one of resolution: how finely patients must be classified before stratification stops adding therapeutic information. This section traces the evidence that immunomodulatory benefit is endotype-dependent, identifies the ceiling reached by current single-axis stratification, and argues that mechanism-based endotyping is a plausible, but as yet untested, prerequisite for matching host-directed agents to patients.

Corticosteroids provide the clearest demonstration that benefit depends on the inflammatory state. In the CAPE COD trial, adjunctive hydrocortisone reduced 28-day mortality among patients with severe CAP admitted to intensive care (11). Pooled and secondary analyses sharpen this signal, indicating that the mortality reduction concentrates in patients with high systemic inflammation, whereas those with lower inflammatory activity derive little benefit and may be exposed to harm from hyperglycemia and immunosuppression (12, 54). These subgroup and pooled findings are hypothesis-generating: they establish that the direction of effect can track a measurable inflammator*y* axis rather than severity alone, but they are not prospectively validated predictive biomarkers, and the primary randomized evidence should be distinguished from the secondary analyses that resolve the interaction (12, 54). Read this way, the corticosteroid record demonstrates heterogeneity of treatment effect and supplies a rationale for predictive enrichment, rather than proof of an established endotype-to-drug matching paradigm. This principle is the foundation on which precision immunomodulation is built, and it invites the question of how reliably such subgroups can be defined in advance.

The corticosteroid record also shows why timing and selection, not the drug's potency, decide the outcome. Earlier trials that enrolled unselected patients produced inconsistent results, and a consistent mortality benefit emerged only when analyses focused on those with marked systemic inflammation (12). Later work refined this further, indicating that patients whose inflammation was already resolving drew no benefit, whereas those with persistent hyperinflammation did (54). The lesson generalizes beyond steroids: an immunomodulator helps when it reaches the right host at the right phase, and the task of a stratification scheme is to identify that host and that phase (11).

Data-driven approaches have answered that question in part by resolving CAP into recurring subphenotypes. Latent class analyses of trial and cohort data consistently separate a hyperinflammatory class, marked by elevated cytokines, higher creatinine, and worse outcomes, from a less-inflamed class (Figure 1) (13). These subgroups are prognostically consistent across cohorts, yet their value for predicting corticosteroid response has proven inconsistent: an early signal that the hyperinflammatory class benefited preferentially was not reliably reproduced, and validation work concluded that the subgroups do not yet guide steroid therapy (14). More recent multinational work has reproduced two subphenotypes using a small set of routine variables, linked them to distinct metabolomic signatures, and detected an interaction between corticosteroid treatment and mortality, which suggests that the biological information carried by these classes is real even where its therapeutic use remains unsettled (55, 56). Reproducibility across populations, however, is not yet established: subphenotypes derived in one setting can shift with case-mix, etiology, and the variables used to define them, and none has been prospectively validated as a treatment-assignment tool (14, 55).

Translating these subgroups into treatment decisions depends on classifiers that can be deployed at the bedside. Blood transcriptomic and proteomic signatures are being developed to assign patients to immunological states in near real time, mirroring progress in sepsis and acute respiratory distress syndrome, where biologically defined subphenotypes showed differential treatment responses in secondary trial analyses (9). The obstacle is not the existence of biology but its measurement: a signature must be fast, reproducible across sites, and tied to a specific therapeutic choice before it can guide care (55). Until that link is forged, endotyping informs prognosis more than prescription.

The limits of single-axis stratification define the central tension of this field. When classification is pushed beyond broad subgroups, its therapeutic yield falls: within an already hyperinflammatory population, data-driven sub-phenotyping distinguished biologically different clusters but did not modify the response to methylprednisolone (15). Blood-based host-response classifiers intended to assign patients to immunological states remain under development and have not been prospectively coupled to a treatment decision (9). The endotype-to-therapy link therefore remains unproven at its current granularity. A summary inflammator*y* axis such as C-reactive protein separates patients who will or will not benefit from broad anti-inflammatory action; this practical boundary is what we term the CRP ceiling. It cannot specify which effector pathway drives injury in a given patient, or which targeted intervention should follow. Overcoming this ceiling would require endotypes defined by mechanism rather than by aggregate inflammatory magnitude. It also requires attention to the opposite pole, the hypoinflammatory or immunoparalysed patient, in whom further suppression is harmful and the rational goal is to restore rather than restrain effector function (57).

A mechanism-based view of endotypes is reinforced by the wider palette of host-directed agents, whose effects are similarly conditional. Granulocyte-macrophage colony-stimulating factor illustrates the point in both directions. Systemic administration failed to improve outcomes in acute lung injury, whereas local delivery protected in a post-influenza pneumococcal model (58, 59). The GM-CSF data are themselves a cautionary example: the same mediator can be futile or protective depending on route, timing, and host state, and it shows why unselected immunomodulation has so often failed (57). The broader debate over boosting vs. blocking this cytokine turns on the patient's immune state (57). Beyond cytokine modulation, adjunctive host-directed agents have repeatedly failed prospective testing in severe CAP: in the CAPTIVATE trial, tifacogin (recombinant tissue factor pathway inhibitor) was biologically active yet conferred no mortality benefit, a promising sepsis-subgroup signal that did not survive a dedicated CAP trial (60). Simvastatin, aimed at reversing neutrophil dysfunction, improved neutrophil migration and reduced NETosis in older patients with septic pneumonia, a benefit expected only where neutrophil paralysis is the operative defect (61). In the context of multidrug-resistant CAP, where antibiotic options are narrowing, host-directed and non-antibiotic strategies are being pursued explicitly as interventions that do not depend on bacterial susceptibility, which widens the set of choices that a stratification scheme must weigh (62). Each of these agents targets a specific node, so their rational deployment presupposes an endotype that names that node rather than one that reports overall inflammation.

The translational gap that emerges is a broken chain running from biomarker to endotype to drug. Current stratification captures the magnitude of inflammation robustly enough to identify who might benefit from broad suppression, but not the mechanism precise enough to indicate what to give, and the single-target agents best suited to precision are the ones most likely to fail when applied across a mechanistically mixed population. Closing this gap depends on two developments that the following sections address: endotypes resolved to the level of effector pathways such as inflammasome activity, NET burden, and resolution capacity; and interventions able to engage several of these nodes with adjustable timing rather than acting on one mediator in isolation. These mechanism-based endotypes are, at present, a hypothesis: whether the relevant biology partitions into discrete classes or varies along continuous axes is unresolved, and the proposal is offered as a testable organizing scheme rather than a validated taxonomy. Natural products, by virtue of their multi-target pharmacology, are candidates for the second requirement, and human lung organoids offer a route to test endotype-matched hypotheses under controlled, pathogen-specific conditions.

## 4 Natural products as multi-target host-directed immunomodulators

The defining pharmacological property that qualifies natural products as host-directed therapy is multi-target convergence: structurally diverse plant-derived compounds act simultaneously on the effector nodes that drive lung injury in CAP. This property recommends them for a disease whose injury arises from several innate-immune pathways at once, and it reframes a long empirical tradition of herbal use in respiratory illness as a set of mechanistic hypotheses that can now be tested directly. Rather than killing bacteria, flavonoids, polysaccharides, terpenoids, and polyphenols dampen inflammasome output, restrain NF-*κ*B-driven cytokine production, temper neutrophil-derived damage, and shift macrophages toward resolution, often with a more tolerable toxicity profile than single-target synthetic inhibitors. This section organizes that evidence around the mechanistic axes established earlier, sets out the low-toxicity rationale that distinguishes natural products from targeted immunomodulators, surveys the clinical evidence from traditional formulas in pneumonia, and delimits what remains unproven. Because “natural products” is not a pharmacologically homogeneous category, we distinguish three tiers with very different translational requirements: (i) defined small molecules (e.g., andrographolide, baicalin, astragaloside IV, curcumin), for which a single chemical entity and its mechanism can in principle be characterized; (ii) standardized extracts or fractions (e.g., Astragalus polysaccharides), defined by a controlled constituent profile; and (iii) multi-component formulas (e.g., XueBiJing, Lianhua Qingwen, Tanreqing), which contain many constituents whose active principles and interactions are only partly known. Chemical characterization, dose standardization, pharmacokinetic (PK) attribution, batch-to-batch reproducibility, and regulatory pathway differ sharply across these tiers, and evidence quality should be judged within, not across, them.

Across chemical classes, defined small-molecule natural products converge on a shared set of innate-immune targets rather than acting through class-specific mechanisms. Andrographolide, a diterpenoid, protects against endotoxin-induced lung injury by inactivating NF-*κ*B and, in related models, by suppressing NLRP3 inflammasome assembly (20, 63); its water-soluble sulfonate derivative reproduces this effect through combined MAPK and NF-*κ*B inhibition (64). The flavonoid baicalin limits influenza-associated lung inflammation by downregulating TLR7/MyD88 signaling and the downstream NF-*κ*B and cytokine cascade (22), while astragaloside IV suppresses influenza-driven inflammation through the ROS/NLRP3/caspase-1 axis (21). Standardized polysaccharides add a distinct route, with astragalus polysaccharides attenuating lung injury by remodeling the intestinal microbiota along the gut-lung axis (65). Polyphenols such as curcumin exert bimodal control, deactivating NF-*κ*B while activating Nrf2-dependent antioxidant defenses, and can be paired with glycyrrhizin to overcome poor bioavailability (66, 67). The recurrence of NLRP3, TLR4/NF-*κ*B, and Nrf2 as common endpoints across unrelated scaffolds is now documented systematically, and it is this convergence, rather than any single molecule, that defines the class as host-directed (Figure 2) (16, 23). An important caveat qualifies this classification: several of these agents also exert direct antimicrobial or antiviral activity, so a fall in pathogen burden can reduce inflammation without any genuinely host-directed mechanism. We therefore treat an intervention as host-directed only where an immunomodulatory effect is demonstrated independently of pathogen killing, and label agents with plausible direct antimicrobial action as dual host/pathogen-directed; disentangling the two requires experiments that separate host modulation from bacterial clearance (Figure 2).

**Figure 2:**
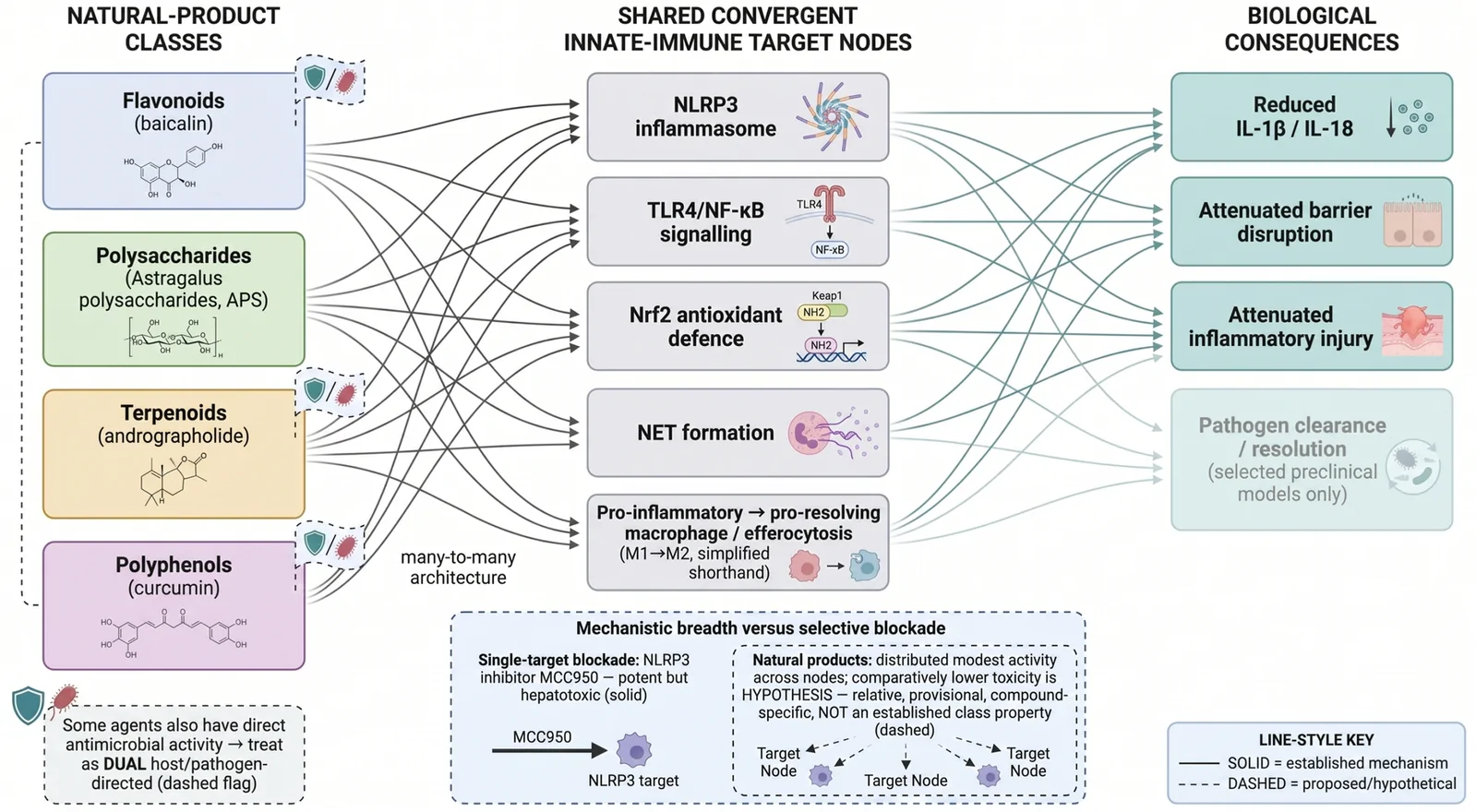
Convergent multi-target actions of natural products on the innate-immune nodes driving lung injury. Structurally diverse natural-product classes, including flavonoids (baicalin), polysaccharides (*Astragalus* polysaccharides, APS), terpenoids (andrographolide), and polyphenols (curcumin), converge on a shared set of innate-immune targets rather than acting through class-specific mechanisms (16, 23). Convergent nodes include the NLRP3 inflammasome, TLR4/NF-*κ*B signalling, Nrf2-dependent antioxidant defence, NET formation, and the pro-inflammatory-to-pro-resolving (M1-to-M2)/efferocytosis programme (20–22, 63, 66, 68). Engagement of these nodes yields the biological consequences shown at right: reduced IL-1β/IL-18 output, attenuated barrier disruption, and attenuated inflammatory injury, with pathogen clearance or resolution effects reported in selected preclinical models. Connecting lines indicate a many-to-many “one agent-multiple targets, one target-multiple agents” architecture. Some agents may also exert direct antimicrobial activity; where this is plausible they are best regarded as dual host/pathogen-directed, and a host-directed effect should be demonstrated independently of pathogen killing. Inset: unlike single-target blockade, exemplified by the NLRP3 inhibitor MCC950 whose potency is offset by hepatotoxicity, natural products are proposed to distribute modest activity across several nodes; their comparatively lower toxicity is a hypothesis that is relative, provisional, and compound-specific rather than an established class property (17). Created in BioRender. Zhang, F. (2026) https://BioRender.com/t74ic7v

The specificity of these actions distinguishes mechanistic host-direction from a nonspecific antioxidant effect. Baicalin acts upstream at pattern recognition, lowering TLR7 and MyD88 expression before the NF-*κ*B cascade engages (22). Astragaloside IV instead intercepts the inflammasome, suppressing the reactive oxygen species that prime NLRP3 and the caspase-1 maturation of IL-1β and IL-18 (21). Astragalus polysaccharides act at a distance, reshaping the intestinal microbiota so that circulating metabolites lower pulmonary inflammatory tone along the gut-lung axis (65). Curcumin combines NF-*κ*B suppression with Nrf2-driven antioxidant induction, a dual action that limits both cytokine output and oxidative damage (66). These are distinct molecular entry points that meet on a shared injury programme, which is the pharmacological basis for treating the class as host-directed rather than simply anti-inflammatory.

The same compounds engage the neutrophil and resolution nodes that separate protective from injurious inflammation, which connects their pharmacology directly to the pathophysiology of severe CAP. Traditional formulas modulate NET formation through defined pathways, as shown for Xuanfei Baidu decoction acting on the CXCL2/CXCR2 axis to limit NET-associated acute lung injury (68). Beyond suppression of inflammatory output, several agents promote the active resolution program, favoring a pro-resolving, efferocytic macrophage phenotype and thereby increasing clearance rather than opposing it, the property that natural-product host-directed therapy shares with endogenous pro-resolving mediators (23, 46). This capacity to attenuate damage while preserving, or even augmenting, pathogen clearance is the central therapeutic claim proposed for natural products in CAP, and it would distinguish them from broadly immunosuppressive strategies that risk impairing host defense. It should be stressed that this claim rests largely on preclinical models; it has not been demonstrated in bacterial CAP with clinical endpoints. The pro-resolving arm is mechanistically specific rather than a vague anti-inflammatory effect. Agents that shift macrophages toward an M2, efferocytic phenotype accelerate the clearance of apoptotic neutrophils and bacterial debris, which removes the stimulus that sustains inflammation instead of merely blunting its output (44, 46).

The multi-target, character of natural products, and its proposed low-toxicity corollary, acquire practical weight when set against the difficulties of single-target immunomodulators. Selective inhibition of one node can be potent but fragile: the NLRP3 inhibitor MCC950, despite compelling preclinical activity, was halted in clinical development after producing hepatotoxicity, and next-generation analogs have encountered further organ-specific toxicity (17). Efforts to repurpose safer agents, such as disulfiram acting on gasdermin D and NLRP3, reflect the same search for tolerable inhibition of this pathway (69, 70). Natural products are hypothesized to approach the problem differently, distributing modest activity across several targets so that no single node is inhibited to the degree that provokes toxicity, and screening low-toxicity NLRP3-active compounds from natural sources has become an explicit strategy on these grounds (16). This distributed-activity argument is a hypothesis, not an established pharmacological rule, and it cuts both ways: polypharmacology can equally broaden off-target effects, drug–drug interactions, and metabolic liabilities, and the single MCC950 example cannot support a class-level safety comparison (17). Natural-product safety is, in fact, compound- and formulation-specific. Herb-induced liver injury is well documented for certain preparations, batch-to-batch compositional variability and contamination or adulteration can introduce hepatic and renal toxicity, and herb–drug interactions (including modulation of cytochrome P450 enzymes and drug transporters) are a material concern when these agents are added to antibiotics and corticosteroids in critically ill patients (18). Any low-toxicity framing is therefore comparative and provisional, and must be established compound by compound rather than assumed for the class. The advantage extends beyond safety to suitability for a multi-node endotype. A response driven by inflammasome activity, NET burden, and resolution failure is more plausibly addressed by an agent that touches all three nodes than by one that silences a single mediator. Conversely, targeted single-node agents retain real advantages: they are titratable, mechanistically attributable, and, where a single causal pathway dominates, may be more precise. The two approaches are therefore best viewed as complementary rather than as competitors, with the choice governed by whether injury is driven by one dominant pathway or by several co-activated nodes.

Formulation and lung exposure are a distinct and frequently neglected requirement, and matching a compound to a pathway on the basis of *in vitro* or animal target activity is insufficient without them. Many natural products are limited by poor solubility and low oral bioavailability, and whether the active constituent (or an active metabolite rather than the parent compound) reaches the alveolus at a concentration and for a duration sufficient to modulate its target is often unknown for the doses used in models (67, 71). Encapsulation, such as curcumin carried in glycyrrhizic-acid nanoparticles, raises pulmonary exposure and illustrates how formulation, systemic vs. inhaled route, and PK/PD behaviour can sharpen or defeat the intended mechanistic match (67). We therefore treat formulation and achievable lung exposure as an explicit fourth layer of the framework developed in Section 5, alongside endotype, target, and compound.

Clinical evidence for natural-product host-directed therapy in pneumonia is concentrated in multi-component formulas used alongside antibiotics, though its quality is uneven (Table 1). The strongest signal comes from XueBiJing injection, which in a multicenter placebo-controlled trial in severe CAP reduced 28-day mortality and shortened mechanical ventilation and intensive-care stay (19). Lianhua Qingwen granule improved symptom resolution in a randomized trial of non-influenza viral pneumonia (71), and meta-analyses of Shufeng Jiedu and Tanreqing as adjuncts to antibiotics report faster resolution of fever, cough, and radiographic infiltrates, with Tanreqing benefit extending to pneumococcal pneumonia specifically (72–74). Evidence mapping across formulas identifies Qingjin Huatan and the injections XueBiJing and Tanreqing as probably beneficial in severe pneumonia while emphasizing the need for larger, higher-quality trials (75). The magnitude of benefit should be read against this quality limit. The XueBiJing trial, the most rigorous example, randomized 675 patients across many centres and reported an absolute mortality reduction of roughly nine percentage points (19), whereas the symptom-level gains pooled for Shufeng Jiedu and related formulas rest on smaller, more heterogeneous studies with wider confidence intervals (72). Several caveats limit how far these trials can be pushed. Many enrolled patients with clinically rather than microbiologically confirmed CAP, so the etiology (bacterial vs. viral or mixed) is often uncertain; the interventions were adjunctive to standard antibiotics, so benefit cannot be attributed to host modulation alone; trials are geographically concentrated and formulations vary; and a meta-analysis of small or heterogeneous studies does not by itself confer high certainty. Crucially, none of these trials was designed around a defined inflammatory endotype, so they demonstrate the clinical feasibility of adjunctive natural-product therapy rather than validation of endotype-matched treatment. Mechanistic and clinical work on Maxing Shigan decoction is notable for spanning both pathogen classes, promoting anti-inflammatory M2 macrophage polarization and modulating PI3 K/AKT signaling in influenza-induced injury and acting through IL-17A/HIF pathways in *P. aeruginosa* pneumonia (76, 77). Of particular relevance to the multidrug-resistant setting, Yinhuapinggan granule is being evaluated as complementary therapy in community-acquired drug-resistant bacterial pneumonia (78). The consistent caveat across this literature is methodological: most trials are small, single-center, or at moderate-to-high risk of bias, so the benefits observed should be read as promising rather than definitive.

**Table 1:** Representative natural products and formulas for host-directed therapy in pneumonia. Entries are annotated by study design, pathogen/trigger, and evidence directness for bacterial CAP, and by a separate certainty/limitations note; “study design” labels the type of study and does not by itself denote certainty. Evidence directness follows a hierarchy from human bacterial CAP (most direct) through human CAP of mixed/unspecified etiology, bacterial infection models, viral pneumonia, and sterile/LPS or other acute-lung-injury models, to *in vitro* mechanistic data (least direct). Most listed clinical trials used interventions adjunctive to standard antibiotics and enrolled patients with clinically rather than microbiologically confirmed CAP, so benefit cannot be attributed to host modulation alone. Registered or published trial protocols without outcome data are labeled as such and do not constitute efficacy evidence.

Agent (compound/formula)	Class	Molecular target(s)	Model	Pathogen/trigger	Evidence level	CAP relevance	Certainty/limitations	Ref
Andrographolide	Terpenoid (diterpenoid)	NF-*κ*B, NLRP3	Mouse ALI	LPS	Animal	Indirect (endotoxin)	Preclinical only; single small molecule; no CAP data	(20, 63)
Andrographolide sulfonate	Terpenoid derivative	MAPK/NF-κB	Mouse ALI	LPS	Animal	Indirect (endotoxin)	Preclinical only; derivative	(64)
Baicalin	Flavonoid	TLR7/MyD88, NF-κB	Mouse	Influenza A	Animal	Viral pneumonia	Preclinical; viral not bacterial	(22)
Astragaloside IV	Terpenoid saponin	ROS/NLRP3/caspase-1	Mouse/cell	Influenza	Animal/*in vitro*	Viral pneumonia	Preclinical; viral model	(21)
*Astragalus* polysaccharides	Polysaccharide	Gut–lung axis	Mouse ALI	LPS	Animal	Indirect (endotoxin)	Preclinical; indirect (gut-lung); standardized extract	(65)
Curcumin	Polyphenol	NF-κB, Nrf2	Mouse/cell	PM/LPS	Animal/*in vitro*	Indirect (endotoxin)	Preclinical; bioavailability-limited	(66, 67)
Xuanfei Baidu	Formula	CXCL2/CXCR2, NET formation	Mouse ALI	LPS/IgG–IC	Animal	Indirect (NET)	Preclinical; multi-component (standardization)	(68)
Maxing Shigan	Formula	PI3 K/AKT; IL-17A/HIF	Mouse	Influenza; *P. aeruginosa*	Animal	Bacterial + viral	Preclinical; includes a bacterial model	(76, 77)
XueBiJing	Multi-component formula (injection)	Multi-target anti-inflammatory (broad)	Multicentre RCT	Severe CAP(clinical)	RCT (reduced 28-day mortality)	Direct: human CAP (etiology often mixed/unconfirmed)	Adjunctive to antibiotics; not endotype-designed; feasibility, not mechanistic validation	(19)
Lianhua Qingwen	Formula	Multi-target	RCT	Non-influenza viral pneumonia	RCT	Viral pneumonia	Adjunctive; viral not bacterial CAP	(71)
Shufeng Jiedu	Formula	Anti-inflammatory/immunomodulatory	Meta-analysis (adjuvant)	CAP	Meta-analysis	Direct CAP	Small/heterogeneous trials; meta-analysis ≠ high certainty; adjunctive	(72)
Tanreqing	Formula (injection)	Anti-inflammatory	SR/meta-analysis	CAP; *S. pneumoniae*	Meta-analysis	Direct bacterial CAP	Moderate-to-high risk of bias; adjunctive	(73, 74)
Yinhuapinggan	Formula	Anti-inflammatory/antiviral	RCT	MDR bacterial CAP	RCT protocol	MDR bacterial CAP	Protocol only; no efficacy/safety data reported	(78)

ALI, acute lung injury; CAP, community-acquired pneumonia; IgG-IC, IgG immune complex; LPS, lipopolysaccharide; MDR, multidrug-resistant; NET, neutrophil extracellular trap; PM, particulate matter; RCT, randomized controlled trial; SR, systematic review. Bold entries mark the strongest and most CAP-relevant evidence.

Beyond the completed trials above, some of the most directly relevant work to the multidrug-resistant setting is still prospective. Yinhuapinggan granule, for example, is being evaluated as complementary therapy in community-acquired drug-resistant bacterial pneumonia, but the published record to date is a randomized, double-blind, controlled trial protocol rather than an efficacy study; it demonstrates planned investigation and should not be read as evidence of treatment benefit or safety until outcome data are reported (78). We flag such registered or published protocols explicitly to keep planned investigation separate from completed efficacy evidence (Table 1).

Two limitations bound the current evidence and set the agenda for the sections that follow. First, mechanistic depth is concentrated in viral pneumonia and endotoxin-driven acute lung injury models rather than in bacterial CAP with clinical endpoints, so the pathway-level claims and the outcome-level trials are not yet joined by studies that link a defined natural-product mechanism to a bacterial-CAP result (23). Second, the multi-target property that underlies the low-toxicity advantage is also a source of mechanistic ambiguity, because polypharmacology complicates attribution of effect, standardization of multi-component formulas, and pharmacokinetic characterization, and no natural product has been matched prospectively to a defined inflammatory endotype. As emphasized above, the low-toxicity framing is comparative and provisional rather than absolute (18). The low-toxicity framing is comparative rather than absolute, since several natural products carry dose-dependent hepatic or renal liability, batch-to-batch compositional variability, and potential herb-drug interactions that must be characterized before clinical use. Standardization is the practical bottleneck. A multi-component formula that shows benefit in one preparation may not reproduce it in another unless the constituent profile is controlled, and pharmacokinetic behaviour, including the exposure actually reached in the lung, is often uncharacterized for the doses used in models (71). These gaps identify the two requirements that motivate the remainder of the review: a framework that assigns multi-target agents to the endotypes they are best suited to correct, and a human-relevant platform capable of resolving their pathogen-specific, mechanism-level effects before clinical testing.

## 5 Matching natural products to endotypes: a proposed target-endotype framework

The proposal advanced here is a multi-layer mapping that links each candidate inflammatory endotype to the effector pathway driving its injury and, in turn, to natural products whose multi-target footprint covers that pathway, with formulation and lung exposure as a fourth translational layer. Its purpose is to convert the polypharmacology of natural products from an interpretive liability into a matching asset, and to add therapeutic resolution precisely where classification by a single inflammator*y* axis reaches its ceiling. It is offered as a hypothesis-generating scaffold to structure future experiments, not as a validated clinical algorithm. The framework reframes the earlier evidence: the mechanistic nodes of CAP immunopathology become the coordinates of the candidate endotype, the conditional benefit of immunomodulation becomes a matching problem, and the breadth of natural-product activity becomes a proposed means of addressing it. What follows specifies the mappings, argues where the scheme might outperform single-axis stratification, incorporate the pathogen dimension, and states the conditions under which it can be tested (Figure 3).

**Figure 3:**
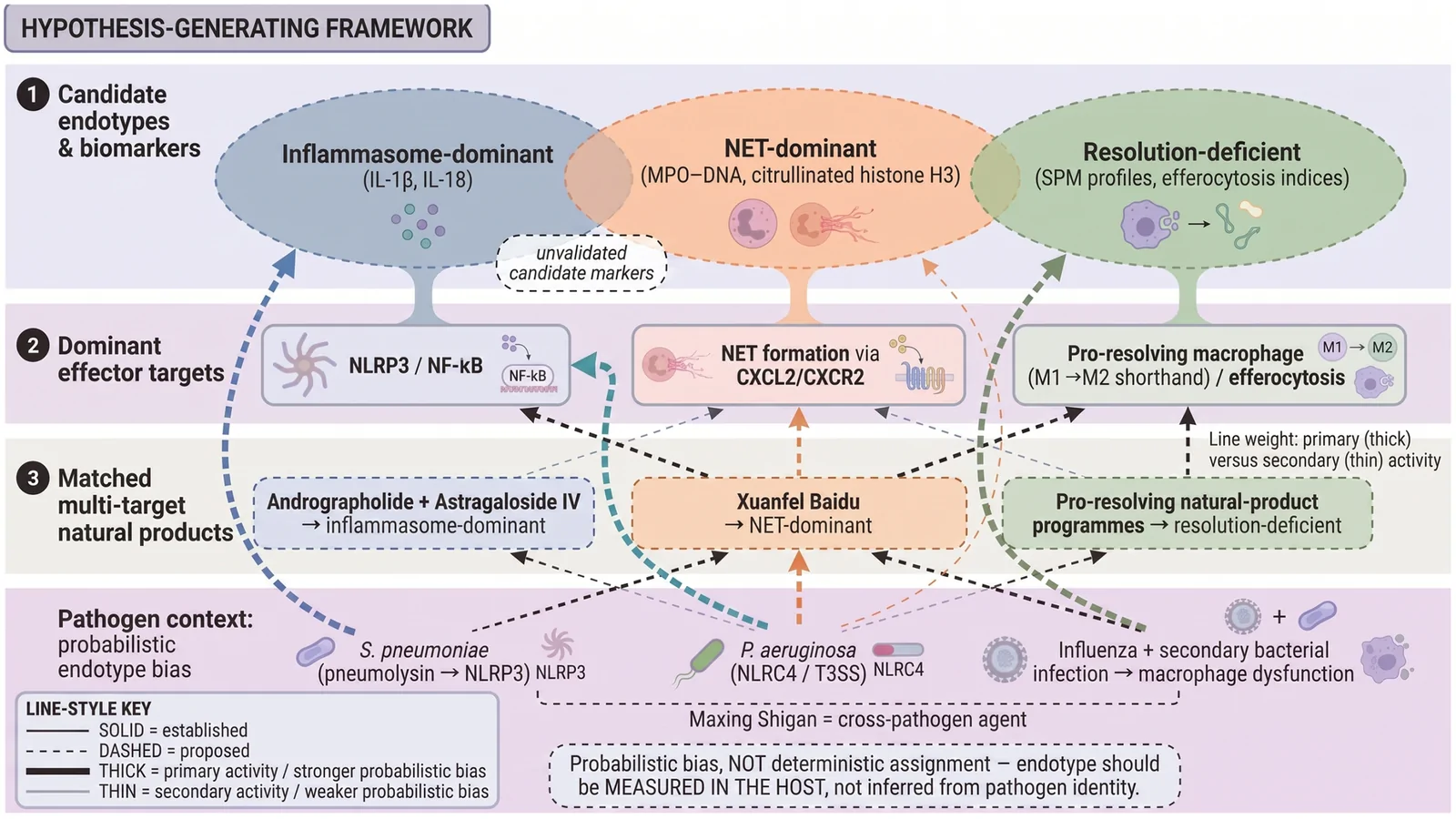
An endotype-target-natural product framework matched along an additional pathogen axis. The proposed three-layer framework links each candidate inflammatory endotype to the effector pathway driving its injury and, in turn, to natural products whose multi-target footprint covers that pathway. Layer 1 (endotypes and candidate biomarkers): inflammasome-dominant (IL-1β, IL-18), NET-dominant (MPO-DNA, citrullinated histone H3), and resolution-deficient (specialized pro-resolving mediators, efferocytosis indices) states, defined by pathway-level readouts rather than aggregate inflammatory magnitude (13, 15). These states are proposed and potentially overlapping rather than validated or mutually exclusive, and the listed biomarkers are unvalidated candidates. Layer 2 (dominant effector targets): NLRP3/NF-*κ*B, NET formation via CXCL2/CXCR2, and the pro-resolving macrophage (M1-to-M2)/efferocytosis programme. Layer 3 (matched multi-target natural products): andrographolide and astragaloside IV for the inflammasome-dominant state, Xuanfei Baidu for the NET-dominant state, and pro-resolving natural-product programmes for the resolution-deficient state; line weight denotes primary vs. secondary activity (21, 46, 63, 68). A horizontal pathogen axis shows that the same endotype is probabilistically biased, not deterministically assigned, by the causative organism: *S. pneumoniae* (pneumolysin→NLRP3), *P. aeruginosa* (NLRC4/T3SS), and influenza with secondary bacterial infection, with Maxing Shigan illustrating a cross-pathogen agent (47, 49, 76, 77). Endotype should be measured in the host rather than inferred from pathogen identity. The framework advances multi-target coverage as tolerant of imperfect classification, stated as a testable hypothesis rather than an established result. Created in BioRender. Zhang, F. (2026) https://BioRender.com/dew32ob

The first layer redefines endotypes by dominant effector mechanism rather than by aggregate inflammatory magnitude. Where current stratification separates hyperinflammatory from hypoinflammatory patients using summary markers such as C-reactive protein, a mechanism-based scheme distinguishes an inflammasome-dominant state, a NET-dominant state, and a resolution-deficient state, each defined by pathway-level readouts rather than by total cytokine load (13). These are proposed mechanistic states, or candidate endotypes, not validated categories. They are unlikely to be mutually exclusive or static: a single patient may simultaneously show inflammasome activation, NET excess, and impaired resolution, and the balance may shift over the course of illness. The scheme is therefore better conceived as a set of continuous, co-varying axes (or a multidimensional score) than as three discrete boxes, and “dominance” denotes the currently prevailing axis rather than an exclusive category. Where more than one axis is elevated, assignment would follow the highest-weighted readout while retaining the others as secondary targets, and a multi-target agent is attractive precisely because it need not resolve this ambiguity perfectly. This refinement is motivated directly by the observation that sub-phenotyping within a hyperinflammatory population failed to modify treatment response, a failure consistent with the possibility that the operative distinction is not more or less inflammation but which effector arm predominates (15). Grounding endotypes in the inflammasome, NET, and resolution axes established as the interventable core of CAP injury would convert a descriptive severity gradient into a set of actionable mechanistic categories (16). Each state carries candidate, measurable readouts that could operationalize assignment: an inflammasome-dominant state through IL-18, IL-1β, and caspase-1 activity, a NET-dominant state through circulating myeloperoxidase-DNA complexes, citrullinated histone H3, and cell-free DNA, and a resolution-deficient state through specialized pro-resolving mediator profiles and indices of macrophage efferocytosis (Table 2). These readouts are candidates rather than validated assays, and each carries substantial interpretive and feasibility limits. Several are nonspecific or not lung-specific: circulating NET markers reflect systemic as well as pulmonary neutrophil activity; caspase-1 activity and cell-free DNA rise in many inflammatory states; and specialized pro-resolving mediator and efferocytosis measurements are technically demanding, assay-dependent, and unstable. For each, the sampling compartment (plasma, serum, or bronchoalveolar lavage), analytical method, timing relative to illness onset, turnaround time compatible with a bedside decision, and validation status remain to be defined (Table 2). No thresholds have been established. A workable scheme will therefore read a minimal composite panel together rather than rely on any single marker, with thresholds calibrated against outcome rather than against healthy controls, and biomarker feasibility is a precondition for, not a detail of, the framework.

**Table 2:** Proposed mechanistic endotypes: dominant pathways, candidate biomarkers, matched natural products, and organoid readouts. Biomarkers are candidates rather than validated assays; a workable scheme reads a small panel together, with thresholds calibrated against outcome. Listed organoid readouts assume the neutrophil/macrophage co-culture and chip-standardization augmentations described in Figure 4.

Mechanistic endotype	Dominant pathway	Candidate biomarkers	Matched natural product(s)	Organoid readout (with augmentation)	Ref
Inflammasome-dominant	NLRP3/NLRC4 → caspase-1/GSDMD	IL-18, IL-1β, caspase-1 activity	Andrographolide, astragaloside IV	Epithelial pyroptosis/IL-1β (± monocyte co-culture)	(6, 32, 33, 35)
NET-dominant	NET formation/clearance imbalance	MPO–DNA complexes, citrullinated histone H3, cell-free DNA	Xuanfei Baidu (CXCL2/CXCR2) ± DNase	Neutrophil co-culture NET quantification	(7, 37, 40, 68)
Resolution-deficient	Deficient M1→M2/efferocytosis/SPM	SPM profiles, efferocytosis index	SPM-mimetic natural products, resolvin-pathway agents	Macrophage co-culture efferocytosis/polarization	(44, 46)

GSDMD, gasdermin D; MPO, myeloperoxidase; NET, neutrophil extracellular trap; SPM, specialized pro-resolving mediator.

The second layer matches each candidate mechanistic endotype to natural products whose activity spans its driving pathway. An inflammasome-dominant endotype is addressed by compounds with documented NLRP3 and NF-*κ*B activity, such as andrographolide and astragaloside IV, which suppress caspase-1-dependent cytokine maturation while leaving pathogen recognition intact (21, 63). A NET-dominant endotype maps to agents that restrain trap formation through defined signaling, illustrated by Xuanfei Baidu decoction acting on the CXCL2/CXCR2 axis, a mechanism complementary to enzymatic degradation of extracellular chromatin (68). A resolution-deficient endotype calls for agents that promote a pro-resolving, efferocytic macrophage phenotype, the program shared by natural products and endogenous pro-resolving mediators, restoring clearance rather than adding suppression (44, 46). Because most formulas engage several of these nodes at once, the mapping is not one-to-one but weighted, assigning each agent to the endotype its activity profile best covers while retaining secondary activity against co-existing pathways. The mechanistic evidence underlying these assignments is largely preclinical and, for several agents, derived from non-CAP models, so the matches are candidate pairings to be tested rather than recommendations for use.

A hypothetical scenario shows how the mapping would operate. Consider a patient with severe pneumococcal CAP and high systemic inflammation, whose pneumolysin-driven disease may bias the response toward the inflammasome axis (47). The framework would assign this patient to an inflammasome-dominant endotype, read out by elevated IL-18 and caspase-1 activity, and match a compound with documented NLRP3 and NF-*κ*B activity such as andrographolide or astragaloside IV (21, 63). A patient whose injury is dominated by neutrophil traps would instead be directed toward an agent that limits NET formation through defined signalling, illustrated by Xuanfei Baidu decoction acting on the CXCL2/CXCR2 axis (68). A third patient, in whom inflammation has entered a protracted, resolution-deficient phase, would be matched to agents that promote a pro-resolving, efferocytic macrophage phenotype, the programme shared by natural products and specialized pro-resolving mediators, so that clearance is restored rather than further suppressed (44, 46). The same logic extends to formulas already tested in severe CAP, where a multi-target agent such as XueBiJing could be evaluated prospectively in the endotype its activity best fits rather than across an undifferentiated population (19). This is the step that current trials omit, and the step the framework is designed to supply.

Designing a trial around this framework would invert the usual sequence, and specifying it in falsifiable terms is essential if the framework is to be scientifically useful. Rather than testing one agent across all comers, an endotype-stratified design would first assign patients to a mechanistic state using a prespecified biomarker panel and a predefined assignment algorithm, then randomize within each state to the matched natural product or to placebo To test the core matching hypothesis, the design should specify in advance what result would falsify it (for example, no treatment-by-endotype interaction) and should include: prospective endotype assignment before randomization; a comparison of matched vs. deliberately unmatched treatment; a mechanistic co-primary endpoint (the targeted effector readout) alongside a clinical outcome; a bacterial-clearance and safety endpoint to confirm that host modulation does not impair pathogen control; a formal treatment-by-endotype interaction test; temporal sampling to capture endotype transitions; and external validation of both the assignment algorithm and the effect (11, 48). This borrows the predictive-enrichment logic that clarified corticosteroid benefit, and it would test the framework's core claim, that matching improves on unselected treatment, rather than assume it (11).

The framework's potential advantage over single-axis stratification lies exactly at the ceiling that limits current practice. When injury is driven by more than one effector arm simultaneously, a stratification scheme built on a single inflammator*y* axis cannot indicate which pathway to target, and a single-target drug silences only one node while the others continue to damage the lung. A multi-target agent covers co-activated pathways in one intervention, so its benefit would not depend on resolving the endotype to a single mechanism, and it should be more tolerant of the imperfect classification that is unavoidable when biomarkers are limited, a property we advance as a testable hypothesis rather than an established result. This property is proposed to address the plateau at which finer sub-phenotyping stopped adding predictive value, because the therapeutic breadth of the agent, rather than the precision of the classifier, would absorb residual mechanistic heterogeneity (15). The same breadth might mitigate the toxicity that constrains potent single-target inhibition, since coverage is achieved by distributed modest activity rather than by maximal blockade of one node (17), although, as noted, this depends on the safety of each specific agent (85).

Endotype is co-shaped by the pathogen, so the matching scheme must operate along an organism axis as well as an immunological one. The dominant effector state is biased by virulence strategy: pneumococcal pneumolysis tends to bias the response toward NLRP3, *P. aeruginosa* toward NLRC4-driven pyroptosis modulated by its exotoxins, and antecedent influenza toward macrophage dysfunction and secondary bacterial susceptibility (47, 49). These are probabilistic modifiers rather than deterministic assignment rules: the same organism can produce different dominant states across patients depending on host and disease factors, so endotype should be measured in the host rather than inferred from the identity of the pathogen. Natural products that act across pathogen classes therefore fit naturally into the framework, as shown by Maxing Shigan decoction, which promotes anti-inflammatory M2 macrophage polarization in influenza-induced injury and acts through IL-17A/HIF signaling in *P. aeruginosa* pneumonia, matching two pathogen-defined endotypes through partly distinct mechanisms (76, 77). This organism dimension is most consequential in multidrug-resistant CAP, where the inability to rely on bactericidal therapy makes endotype-matched host modulation a potentially valuable adjunctive strategy, particularly when antimicrobial options are constrained, and where agents that combine host-directed and pathogen-directed activity are actively sought (62). Even here, host-directed therapy is intended to complement rather than replace antimicrobial treatment and source control; bacterial control remains essential in MDR disease.

As a scaffold the framework is hypothesis-generating rather than validated, and its requirements define a concrete research program. Operationalizing the first layer depends on pathway-level endotype biomarkers that report inflammasome activity, NET burden, and resolution capacity at the bedside, which do not yet exist in deployable form. Operationalizing the second depends on characterized multi-target footprints for candidate natural products, so that assignment to an endotype rests on quantified activity rather than on inference from heterogeneous models. A third depends on defined formulation and lung-exposure data, so that a mechanistic match is not defeated by a compound that never reaches its target at an effective concentration. Testing the combined scheme requires a system that can vary endotype, compound, and pathogen under controlled conditions, and read out human, mechanism-level responses before clinical evaluation. Such testing must also guard against two pitfalls: over-fitting a flexible multi-target agent to *post hoc* categories, and the standardization problems inherent to multi-component formulas. Human lung organoids, capable of reconstructing pathogen-specific epithelial responses and of screening natural-product activity, are one platform, among complementary human systems, on which these requirements can begin to be met, and they are the subject of the section that follows.

## 6 Human lung organoids to dissect and screen host-directed therapy

Human lung organoids occupy the experimental space between two-dimensional cell lines and animal models, reconstructing airway and alveolar epithelium with sufficient fidelity to reproduce human host-pathogen interactions and to provide one testbed the testbed the preceding framework requires (24). Their promise for endotype-matched natural-product therapy rests on three capabilities, namely faithful modeling of pathogen-specific epithelial responses, tractable screening of candidate agents, and control over experimental variables that animal models cannot provide. That promise is currently bounded by a limitation that is central rather than incidental to this framework: standard organoids lack the immune and vascular compartments (resident neutrophils and macrophages above all) through which most of CAP injury is transacted, which are precisely the components required to read the inflammasome-, NET-, and resolution-based endotypes the framework depends upon (Figure 4). Current organoids therefore model the epithelial arm of the host response well and its immune arm poorly, and are not, as they stand, a direct validation platform for the proposed endotypes. Nor are they the only relevant human system: human precision-cut lung slices, *ex vivo* lung tissue, primary epithelial–immune co-cultures, and microphysiological lung-on-chip formats can each address parts of this question, and organoids are best described as a promising human-relevant platform among these complementary systems rather than the sole option (24, 79). The value of the platform for this review is specific and bounded: within its epithelial scope, it is a human system in which a candidate agent, a defined pathogen, and a mechanistic readout can be varied together under controlled conditions, provided the immune components are supplied by the augmentations described below.

**Figure 4:**
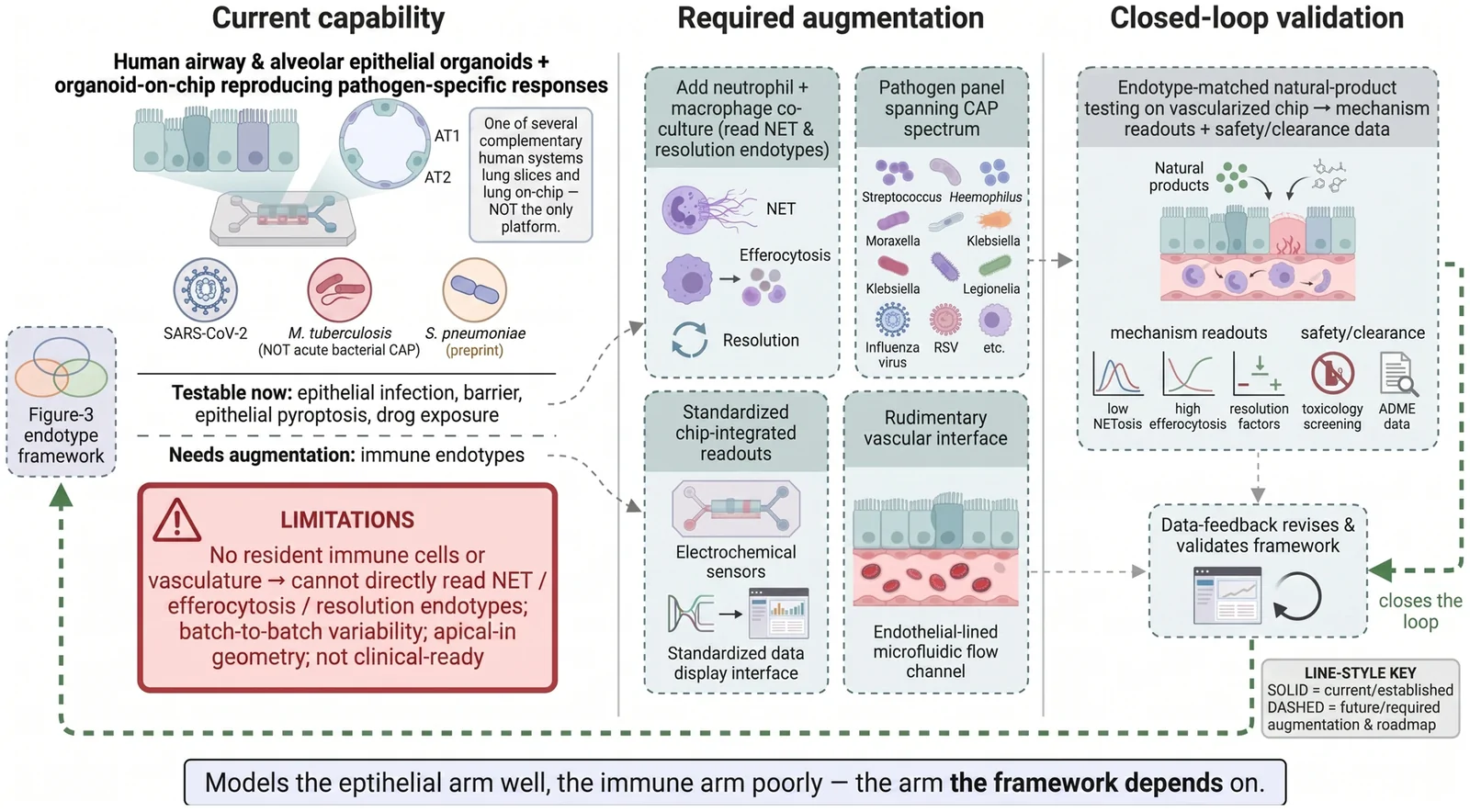
Lung organoid platforms for testing endotype-matched natural-product therapy, and a closed-loop roadmap. Left (current capability): human airway and alveolar epithelial organoids and organoid-on-chip systems reproduce pathogen-specific responses [SARS-CoV-2, *Mycobacterium tuberculosis*, *S. pneumoniae* (preprint)], but current models are limited by absent immune and vascular compartments, batch-to-batch variability, and apical-in geometry, and are not yet clinical-ready (24, 27, 80, 82, 84). Because they lack resident neutrophils and macrophages, current organoids cannot directly report the NET- and resolution-based endotypes central to the framework, and they are one of several complementary human systems (alongside precision-cut lung slices and lung-on-chip) rather than the only relevant platform (79). Middle (required augmentation): adding neutrophil and macrophage co-culture to read NET and resolution endotypes as functional outputs, a pathogen panel spanning the CAP spectrum, standardized chip-integrated readouts, and a rudimentary vascular interface (85, 86). Right (closed-loop validation): endotype-matched testing of natural products on a vascularized chip generates mechanism readouts and safety/clearance data; a data-feedback step then revises and validates the framework, closing the loop back to the Figure 3 scheme (25). The platform models the epithelial arm of the host response well and its immune arm poorly, precisely the arm the endotype framework depends upon, which motivates the augmentations shown. Created in BioRender. Zhang, F. (2026) https://BioRender.com/8ouotfd

Organoids reproduce pathogen-specific host responses across the organisms relevant to CAP. Distal lung organoids derived from adult alveolar progenitors established the platform for respiratory infection, and adult-stem-cell and induced-pluripotent-stem-cell models have since resolved viral tropism and entry mechanisms at the level of individual epithelial lineages (80, 81). Bacterial modeling is less mature but advancing, with emerging organoid-on-chip systems reproducing strain-dependent colonization by *Streptococcus pneumoniae* and identifying cell-type-specific susceptibilities, evidence that currently rests on preprint work (82), and mycobacterial models demonstrating epithelial infection, oxidative-stress responses, and TLR4/NF-*κ*B-associated inflammation within the organoid (27, 83). The bacterial-CAP evidence remains sparse and partly indirect: Mycobacterium tuberculosis is not a model of acute pyogenic bacterial CAP, and the S. pneumoniae organotypic data are preprint rather than peer-reviewed, so neither should be read as showing that pneumococcal organoid modeling is yet mature enough for endotype-matched therapy screening (26, 79, 80). These systems recover some of the pathogen-specific mechanisms that the matching framework treats as endotype coordinates, allowing the organism axis of the scheme to begin to be examined in human tissue rather than inferred from murine infection (Figure 4). The provenance of the epithelium shapes what these models capture. Adult stem cell-derived organoids retain donor airway identity and support long-term culture, whereas induced pluripotent stem cell-derived alveolar organoids offer scalable type 2 cells but require directed differentiation (80, 81). Geometry matters as much as origin, because the receptor-bearing apical surface faces inward in standard cultures and must be everted or exposed on a chip before a luminal pathogen can be introduced efficiently (84).

The same platforms support discovery and validation of host-directed agents. A tiered screening strategy that confirms hits from simpler cultures in lung organoids has been used to identify natural products active against respiratory infection, demonstrating that organoids can be the physiologically relevant filter in a natural-product pipeline (25). In tuberculosis, organoid models have been built explicitly to test host-directed therapies, providing a template for evaluating whether a candidate modulates the host response without compromising epithelial control of the pathogen (27). This screening role is where the framework's second requirement, a characterized multi-target footprint tested under human, pathogen-specific conditions, can begin to be met, within the epithelial limits noted above. Throughput remains a constraint, because organoid assays are slower and more variable than cell-line screens, so a tiered strategy that triages candidates in simpler systems before organoid confirmation is the realistic near-term design (25).

The limitations of current organoids are, for a therapy aimed at the immune response, decisive rather than peripheral, and they set the terms on which the platform can serve the framework. Standard organoids lack resident immune cells and vasculature, so the neutrophil, macrophage, and NET-mediated processes that define the inflammasome-, NET-, and resolution-based endotypes cannot be read directly from an epithelium-only system (24). Batch-to-batch variability constrains quantitative screening, and the apical-in geometry of Matrigel-embedded organoids impedes access to the infected surface, a problem that micro-patterned and organoid-on-chip formats partly resolve (82, 84). Direct evidence in bacterial CAP remains sparse relative to the viral literature, so the platform's fit to the pathogen spectrum of this review is still incomplete (85). The limitations of current organoids are, for a therapy aimed at the immune response, decisive rather than peripheral, and they set the terms on which the platform can serve the framework.

Each limitation has a developing solution, and the relevant systems are best described as future immune-competent organoid or chip platforms. Co-culture systems that add neutrophils and macrophages to epithelial organoids are beginning to reconstruct the cellular interactions that generate NET- and resolution-based endotypes, so that these states can be read as functional outputs rather than inferred (86). Microfluidic organoid-on-chip platforms expose the apical surface and introduce flow, which improves access to the infected epithelium and adds a rudimentary vascular interface (84). Standardized, chip-integrated formats also narrow the batch-to-batch variability that has constrained quantitative screening (85). None of these advances is yet mature for bacterial CAP, but together they chart a plausible path from an epithelium-only model toward a system that can host the immune effectors the framework requires.

Serving the framework therefore requires organoid systems augmented in specific ways rather than adopted as they stand. Co-culture with neutrophils and macrophages would allow the NET and resolution endotypes to be read as functional outputs, pathogen panels spanning the CAP spectrum would let endotype and organism be varied together, and standardized, chip-integrated formats would provide the reproducibility that quantitative endotype-compound testing demands (85, 86). Positioned this way, lung organoids are not a finished validation platform but one of the more credible routes toward one, capable of helping to convert the endotype-target-natural product scheme from a conceptual mapping into a set of testable, human, pathogen-specific hypotheses. The steps needed to realize that role, together with the broader gaps identified across this review, define the outlook considered next.

## 7 Limitations and open questions

Several limitations bound the framework proposed here and define the work needed to test it. The most consequential is the mismatch between where the evidence sits and where the argument points. Mechanistic studies of natural products concentrate in viral pneumonia and endotoxin-driven lung injury, whereas the framework targets bacterial CAP with clinical endpoints, so the pathway-level claims and the outcome-level trials are joined by inference rather than by direct study (23, 71). Closing this gap requires natural-product studies that use bacterial-CAP models and report clinically meaningful outcomes, not cytokine levels alone. The clinical trials that do exist, in turn, rarely report the mechanistic readouts that would connect an outcome to a specific host pathway, so the two literatures pass each other without meeting.

The endotype layer carries its own uncertainty. The mechanistic states proposed here are hypotheses drawn from the biology of inflammasome, NET, and resolution activity, and they have not been validated as discrete, reproducible categories in patients; they may well prove to be overlapping and continuous rather than discrete. Data-driven subphenotyping has so far failed to improve prediction beyond a single inflammator*y* axis (15), which leaves open whether mechanism-based endotypes will fare better, or whether the relevant biology varies continuously rather than in classes. Candidate biomarkers such as IL-18, MPO-DNA complexes, and specialized pro-resolving mediator profiles must be shown to track the states they are meant to index, and their specimen, assay, specificity, timing, and reproducibility characteristics defined, before they can guide therapy.

The natural-product layer must resolve problems of attribution and consistency. Polypharmacology, the property that makes these agents attractive for multi-node endotypes, complicates identification of the active constituent and the mechanism responsible for benefit. Multi-component formulas add batch-to-batch variability and the possibility of herb-drug interactions and compound-specific hepatic or renal toxicity, so standardized, well-characterized preparations, defined lung-exposure data, and explicit safety characterization are a precondition for reproducible study (18). A further ambiguity is that some agents exert direct antimicrobial as well as host-directed effects, so studies must separate host modulation from pathogen killing before an agent is classified as host-directed. Finally, the organoid platform on which validation depends does not yet contain the immune effectors central to the endotypes it is meant to test, and complementary human systems such as precision-cut lung slices and lung-on-chip carry their own limitations (79). Progress on immune co-culture and organ-on-chip systems is real but early, and the framework should be read as a research programme to be tested rather than a scheme ready for the clinic.

A credible translational path would proceed in stages. Immune-competent organoids and complementary human platforms would first establish which natural products modulate which effector states under defined, pathogen-specific conditions, generating the quantified activity profiles the matching layer requires. Biomarker panels validated against outcome would then define the endotypes in patients. Only after these two elements exist could an endotype-stratified trial, designed with the prespecified assignment algorithm and falsifiable interaction test described in Section 5, test whether matched treatment outperforms unselected treatment. Regulatory acceptance of multi-component preparations would in parallel demand standardized manufacturing and defined constituent profiles. None of these steps is trivial, but each is concrete, and together they convert a conceptual framework into a testable clinical hypothesis.

## 8 Conclusion

This review has advanced, as a hypothesis-generating proposal, an endotype-target-natural product framework, testable in human lung organoids and complementary human platforms, that reframes host-directed therapy for community-acquired pneumonia from uniform anti-inflammation toward mechanism-matched, multi-target modulation. The framework's intended value is that it could unify otherwise separate observations: it organizes the injurious host response into candidate effector endotypes, proposes which multi-target natural products might be matched to each, and offers a structure for predicting benefit where a single inflammator*y* axis cannot, though these predictions remain to be tested. We do not claim that the framework is clinically established, that the named agents “correct” these endotypes, or that natural products possess a demonstrated class-level toxicity advantage; each of these is a hypothesis requiring experimental and clinical validation, and host-directed therapy remains adjunctive to, not a replacement for, antimicrobial treatment. Realizing this potential depends on challenges that are layered rather than parallel (Figure 4). At the experimental level, epithelium-only organoids must acquire immune and vascular compartments before they can report the neutrophil- and macrophage-defined endotypes on which the scheme relies. At the level of data integration, pathway-resolved biomarkers of inflammasome, NET, and resolution activity must be linked to quantified compound footprints and to lung-exposure data so that matching rests on measurement rather than inference. Methodologically, two tools are needed to navigate the polypharmacology that makes natural products both attractive and hard to attribute: standardized characterization of multi-component formulas, and artificial-intelligence-guided mapping of target-compound-endotype relationships. These solutions close a loop: immune-competent platforms and integrated, computationally assisted data advance the matched-therapy application, and the translational evidence they generate refines and corrects, and could ultimately validate the conceptual framework from which they derive. Precision host-directed therapy for pneumonia will be defined less by suppressing inflammation than by matching multi-target modulation to the mechanism, the pathogen, and the patient, an aspiration this review frames as a testable research program rather than a current capability.
